# FAM126A interacted with ENO1 mediates proliferation and metastasis in pancreatic cancer via PI3K/AKT signaling pathway

**DOI:** 10.1038/s41420-022-01047-9

**Published:** 2022-05-05

**Authors:** Yongning Li, Ying Li, Jun Luo, Xueqin Fu, Peng Liu, Songbai Liu, Yaozhen Pan

**Affiliations:** 1 College of Clinical Medicine, Guizhou Medical University, Guiyang, Guizhou China; 2 Department of Hepatobiliary Surgery, The Affiliated Hospital of Guizhou Medical University, Guiyang, Guizhou China; 3 Department of Breast Surgery, Guizhou Provincial People’s Hospital, Guiyang, Guizhou China; 4 Department of Hepatobiliary Surgery, The Affiliated Cancer Hospital of Guizhou Medical University, Guiyang, Guizhou China

**Keywords:** Oncogenes, Tumour biomarkers

## Abstract

Pancreatic cancer (PC) is a common digestive system carcinoma with high mortality rate mostly due to aberrant growth and distant metastasis. Current researches demonstrated that Family Sequence Similarities (FAMs) have been involving in tumor development, and which subfamily has the function of promoting or inhibiting tumors and its in-depth molecular mechanism remains unclear. Based on the Gene Expression Omnibus (GEO), the Gene Expression Profiling Interactive Analysis (GEPIA2), we observed that FAM126A is in high expressed level among PC tissues and contributes to worse progression of PC, which was validated by PC tissue microarray. Function assay indicated that overexpression of FAM126A accelerates PC cell proliferation, invasion and migration in vitro, as well as liver cancer metastasis in vivo. Further, we found that FAM126A induces epithelial-mesenchymal transition (EMT), including the downregulation of E-cadherin epithelial marker expression, and the upregulation of N-cadherin, Vimentin, and Snail, mesenchymal marker expression. By co-localization and co-immunoprecipitation assays, we confirmed that FAM126A directly interacts with ENO1, which was a key activator of the PI3K/AKT signaling pathway. Furthermore, ENO1 knockdown reversed cell proliferation, migration, and invasion of PC cells promoted by FAM126A overexpression in vitro and in vivo. In general, these results verified FAM126A is an oncogene interacting with ENO1 in PC by activating PI3K/AKT signaling pathway.

## Introduction

Pancreatic cancer (PC) is predicted to rank second among deadliest cancers in the US by 2030, which has a trend of rapid increase incidence and relative poor prognosis [1–3]. Although, the research on pancreatic cancer continues to advance, its treatment methods are still surgical resection, radiotherapy, chemotherapy, and the treatment effect is not well. Surgical resection remains the best therapeutic approach for PC, and the median survival time in resectable pancreatic ductal adenocarcinoma (PDAC) following surgery is 26 months [4–6]. However, a large part of PC diagnoses occurred at an advanced stage, with patients showing a median survival of only 4.6 months, this part of patients may not be able to receive radical surgery [7–9]. Compared with other tumor diseases, there is no biomarker for early diagnosis and effective treatment of pancreatic cancer. Therefore, there is an urgent need to identify efficient molecular markers for timely diagnosis and precisely therapy, which may also provide insights into the inhibition properties of malignant cells.

Family sequence similarity 126, member A (FAM126A, also termed HYCC1), is found on chromosome 7q15.3. Some of these family members are associated with the development of tumors, including lung, kidney, breast, prostate, and colorectal cancers, as well as esophageal squamous cell carcinoma, playing key roles in EMT [10–15]. Growing evidence suggests epithelial-to-mesenchymal transition (EMT) as an important mechanism of tumor metastasis, invasion, and resistance to chemotherapy [16–18], in which malignant cells lose epithelial features and adhesion with the neighboring tissues, and instead gain stem cell-like properties [19]. Since the EMT signature is common to various cancers [20], numerous studies have focused on explaining and discovering new mechanisms by which EMT regulates cancer progression. However, the function of FAM126A in tumors remains undefined, as well as its relevance to the pathological features of PC.

The PI3K/AKT signaling pathway has been proven to be a key target for cancer promotion a long time ago, and it can play a key regulatory role in tumorigenesis, progression, metabolism, immunity, and so on [21]. Activated AKT phosphorylates downstream factors such as various enzymes, kinases, and transcription factors to regulate cell functions, for example, the EMT process of tumors, rapid proliferation, and distant metastasis [22]. AKT activates IkB kinase (IKK-α), leading to the degradation of NF-κB inhibitor IκB, so that NF-κB is released from the cytoplasm to activate its target genes and promote cell survival [23]. In addition, AKT can inhibit the activity of the proteolytic enzyme caspase-9 or phosphorylate the Bcl-2 family member BAD to prevent the activation of the apoptotic cascade [24]. Family sequence similarity was usually reported as the upstream or downstream regulator influencing and involving in AKT signal pathway [25, 26]. Meanwhile, whether FAM126 regulated the PI3K/AKT pathway mediated pancreatic cancer progress, which needed further exploration.

In the current work, FAM126A expression was assessed in PC samples and cells, and showed upregulation in tumors in comparison with noncancerous adjacent tissue. Next, FAM126A expression was altered in two pancreatic cancer cell lines for exploring the mechanism underpinning FAM126A’s effects on PC. Therefore, the present work suggests FAM126A has a crucial oncogenic function in PC, and may be considered a potential molecular target for tumor treatment.

## Results

### FAM126A is overexpressed in PC tissues and correlates with reduced survival

**Fig. 1:**
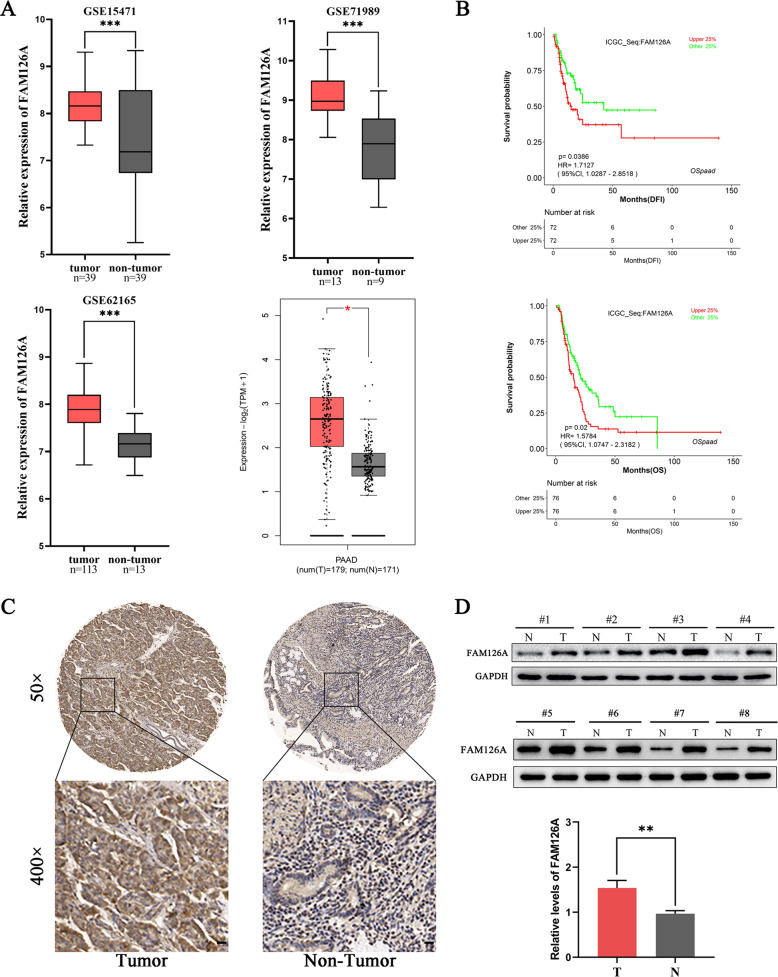
FAM126A is markedly upregulated in pancreatic cancer and shows a positive correlation with reduced survival. **A** FAM126A mRNA amounts are starkly elevated in pancreatic cancer in comparison with noncancerous tissue specimens in GSE15471, GSE71989, GSE62165, and GEPIA2. **B** Kaplan–Meier analysis of the associations of FAM126A levels with OS and DFI in PC cases based on LOGpc. **C** Representative FAM126A images of tissue microarrays for cancer and non-cancerous tissue samples. Magnification, ×50 (×400 for insets). **D** FAM126A is commonly upregulated in PC. Paired PC specimens were examined by immunoblot. *P < 0.05 **P < 0.01, ***P < 0.001.

To determine FAM126A amounts in PC, we firstly analyzed the GEO database and the Gene Expression Profiling Interactive Analysis (GEPIA2) dataset. Three GEO (GSE15471, GSE11838, and GSE16515) and GEPIA2 datasets encompassing PC and adjacent non-cancerous tissue samples were extracted. In all datasets, FAM126A was markedly upregulated in PC in comparison with non-cancerous tissue specimens (Fig. 1A). In addition, to evaluate FAM126A’s prognostic value in PC, we analyzed the associations of FAM126A gene expression with disease-free interval (DFI) and overall survival (OS) using LOGpc datasets. Then, the Kaplan-Meier Plotter analysis proved that overexpression of FAM126A was related with poor OS and DFI in patients with PC (Fig. 1B). Comparing PC and adjacent non-cancerous tissue samples from 49 PC cases, FAM126A protein amounts were elevated in PC tissue samples (Fig. 1C) based upon Immunohistochemical (IHC) staining. Also, Western blot showed higher FAM126A protein amounts in PC specimens in comparison with adjacent non-noncancerous tissue specimens in randomly selected 8 pairs of PC samples (Fig. 1D).

### FAM126A promotes cell proliferation, migration, and invasion

**Fig. 2:**
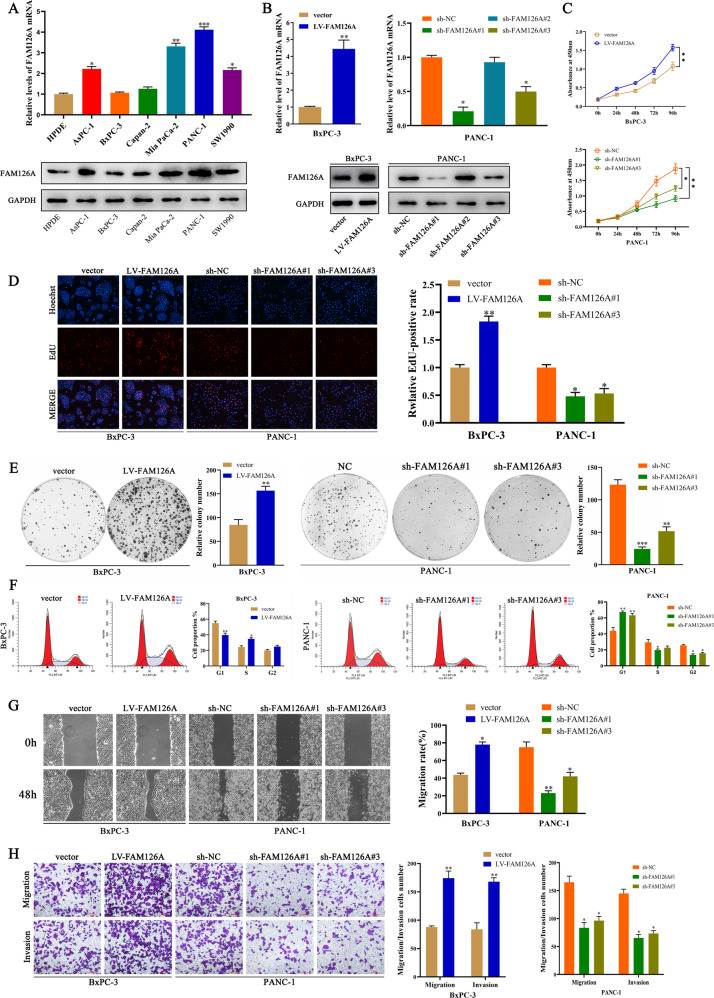
FAM126A enhances proliferation, migration, and invasion in PC cells. **A** FAM126A amounts in six PC cell lines and noncancerous pancreatic ductal epithelial (HPDE) cells. FAM126A mRNA and protein amounts were assessed by quantitative reverse transcriptase (qRT-PCR, upper panel) and immunoblot (lower panel) in AsPC-1, BxPC-3, CaPAN-2, Mia PaCa-2, PANC-1, and SW1990 cells. Relative FAM126A gene expression was normalized to GAPDH. **B** Overexpression of FAM126A in BxPC-3 cells, and knockdown FAM126A in PANC-1 cells. Cells were transfected with lentiviruses to generate BxPC-3-vector/LV-FAM126A and PANC-1-NC/sh-FAM126A#1/sh-FAM126A#3 stable cell lines. FAM126A mRNA (upper panel) and protein (lower panel) amounts were detected as described in (**A**). **C** Cell proliferation assessed with CCK-8 in presence or absence of FAM126A silencing in BxPC-3 (upper panel) or PANC-1 (lower panel) cells. **D**, **E** FAM126A’s effects in clonogenic and EdU assays in PC cells. Representative images and quantitation are depicted in left and right panels, respectively. **F** Cell cycle analysis after FAM126A silencing and overexpression in BxPC-3 and PANC-1 cells. Representative images and quantitation are depicted in left and right panels, respectively. **G** The wound healing assay was carried out to evaluate migration in FAM126A-treated BxPC-3 and PANC-1 cells following scratching for 48 h. **H** The transwell assay evaluated cell migration in FAM126A-treated BxPC-3 and PANC-1 cells. *P < 0.05 **P < 0.01, ***P < 0.001.

To assess whether FAM126A controls PC cell growth, FAM126A amounts were first examined in six PC cell lines, including AsPC-1, BxPC-3, Capan-2, Mia PaCa-2, PANC-1 and SW1990 cells, as well as in noncancerous pancreatic HPDE cells. Western blot and qRT-PCR revealed FAM126A was upregulated in PANC-1 cells compared with HPDE cells, while BxPC-3 amounts in PC cells were lower (Fig. 2A). We performed the following steps to investigate the relationship between FAM126A expression and PC cell proliferation. First, in order to further confirm the effect of FAM126A on PC cells, a FAM126A-lentivirus was constructed and verified for overexpression or knockdown by Western blot (Fig. 2B). Next, CCK-8 assay revealed FAM126A silencing reduced PANC-1 cell proliferation. Consistent with the results of FAM126A knockdown, overexpression of FAM126A increased the viability of BxPC-3 cells (Fig. 2C). Additionally, in comparison with controls, knockdown of FAM126A in PC cells could reduce clonogenicity and proliferation in these cells in EdU incorporation assay, but the colony-formation and proliferation abilities of BxPC-3 cells overexpressing FAM126A were higher than those of the control group (Fig. 2D, E). The above results were also confirmed by cell-cycle analysis that showed a much higher proportion of PC cells in the G1 phase with S-phase reduction after FAM126A silencing, overexpression of FAM126A will have the opposite effect (Fig. 2F). Jointly, the above findings suggested FAM126A played a key regulatory role in PC cell proliferation. To further clarify the roles of FAM126A in PC cell migration and invasion, wound-healing and transwell assays were carried out to examine BxPC-3 and PANC-1 cells. The results revealed FAM126A expression level affected the motility of BxPC-3 and PANC-1 cells. FAM126A-depleted cells showed markedly reduced wound healing ability compared with control cells (Fig. 2G). Transwell migration experiments also confirmed this inhibition. The migration rate was starkly decreased in PANC-1 cells with FAM126A knockdown in comparison with the control group (Fig. 2H). Conversely, FAM126A overexpression remarkably increased wound healing and migratory abilities in BxPC-3 cells.

Next, FAM126A’s effect on the metastatic ability of BxPC-3 and PANC-1 cells was examined by the invasion assay. In comparison with the negative control group, FAM126A silencing significantly reduced the cell invasion rate, while FAM126A overexpression increased the invasive ability of cells. These data suggested FAM126A may enhance migration and invasion in PC cells.

### FAM126A regulates the expression of cell cycle and PI3K/AKT signaling

**Fig. 3:**
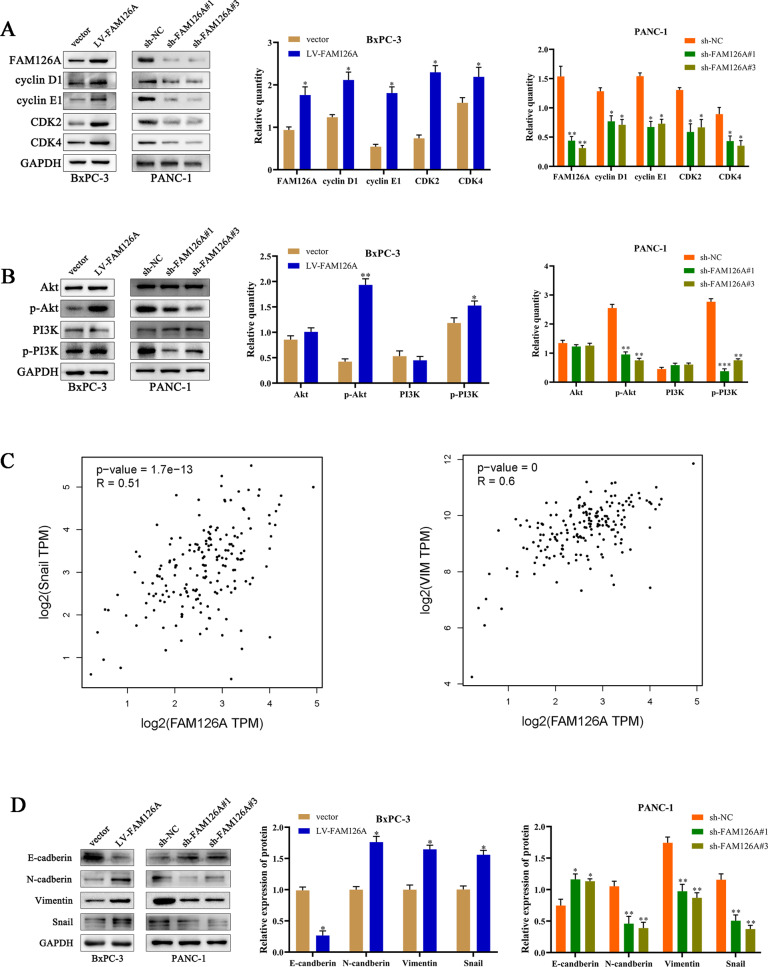
FAM126A induces PC progression. **A** The amounts of cell cycle-related proteins in FAM126A-silenced or overexpressing cells were examined by immunoblot, with GAPDH for normalization. **B** Phosphorylated PI3K and Akt amounts in PANC‑1 and BxPC‑3 cells were examined by immunoblot. **C** In GEPIA2, FAM126A was positively correlated with the EMT-associated transcription factor Snail (*R* = 0.51, *P* < 0.001) and Vimentin (*R* = 0.6, *P* < 0.001) in *P*C tissue samples. **D** Mesenchymal biomarkers, i.e., N-cadherin, Vimentin, and Snail, were downregulated significantly in FAM126A knockdown cells, while the epithelial marker E-cadherin was upregulated. FAM126A overexpression downregulated E-cadherin, while increasing N-cadherin, Vimentin, and Snail amounts. The internal control was GAPDH. *P < 0.05 **P < 0.01, ***P < 0.001.

Furthermore, cell cycle modulators were examined, including Cyclin D1, Cyclin E1, CDK2, and CDK4, which were all downregulated by FAM126A knockdown and upregulated after overexpression of FAM126A (Fig. 3A). To further understand the mechanism of FAM126A in PC, the levels of p-PI3K, PI3K, and p-AKT, AKT were detected. As a result, knockdown of FAM126A remarkably reduced phosphorylated PI3K and AKT amounts, while leaving total protein amounts intact. Then, FAM126A overexpression in BxPC-3 cells have the opposite effect (Fig. 3B). The above findings indicate FAM126A may be an upstream effector of PI3K/AKT signaling in PC and is essential for cell cycle.

### FAM126A regulates EMT

Correlation analysis in GEPIA2 demonstrated FAM126A was positively correlated with Snail (*R* = 0.51, *P* < 0.001) and Vimentin (*R* = 0.6, *P* < 0.001) in PC tissue samples (Fig. 3C). Because EMT is involved in cell motility (12), whether EMT controls FAM126A-associated alteration of migration and invasion was examined. E-cadherin, N-cadherin, Vimentin, and Snail represent EMT hallmarks (16). E-cadherin, N-cadherin, Vimentin, and Snail protein amounts in BxPC-3 and PANC-1 cells were assessed by immunoblot. The mesenchymal biomarkers N-cadherin, Vimentin, and Snail were downregulated significantly upon FAM126A knockdown, while the epithelial marker E-cadherin was upregulated. Conversely, overexpression of FAM126A reduced the expression of E-cadherin, while upregulating N-cadherin, Vimentin, and Snail (Fig. 3D). These data validated FAM126A’s inductive function in EMT.

### Effect of FAM126A silencing on tumor progression in vivo

**Fig. 4:**
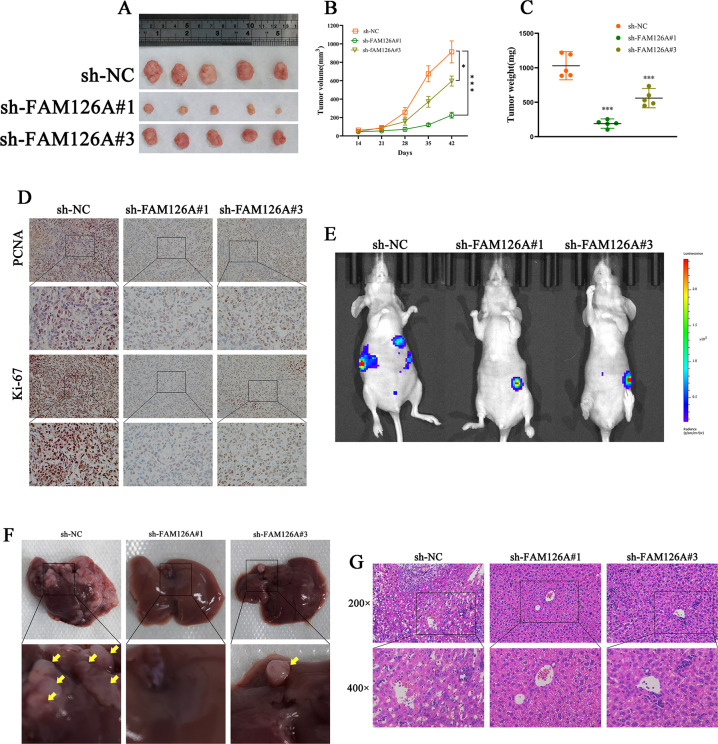
Effects of FAM126A on PC cell proliferation, migration, and invasion in vivo. **A** sh-NC, sh-FAM126A#1, and sh-FAM126A#3 cells were administered by the subcutaneous route to three groups of mice for xenograft model establishment. After 6 weeks, xenografts were extracted and imaged. **B** Tumor volumes were assessed weekly for 42 days. **C** Upon euthanasia, tumors were weighed. **D** IHC showing Ki67 and PCNA protein amounts in tumor samples. **E** Liver metastatic tumor model establishment was performed via injection of PC stable cell suspensions into the mouse spleen. **F** Representative images of liver specimens. Arrow, metastatic tumor. **G** H&E staining of liver metastatic nodules in various groups. *P < 0.05 **P < 0.01, ***P < 0.001.

To assess the impact of FAM126A silencing in vivo, PANC-1-NC and PANC-1-sh-FAM126A cells were transplanted into nude mice. We found that the downregulation of FAM126A significantly inhibited tumor growth. At 42 days after transplantation, FAM126A silencing significantly reduced tumor weight (Fig. 4A–C). Moreover, FAM126A knockdown downregulated the proliferation-related genes Ki-67 and PCNA in xenografts (Fig. 4D), confirming FAM126A as an oncogene in PC. Next, PC stable cell suspension was injected into the spleen of mice to establish a model of liver metastases. After 4 weeks, the ability to metastasize was assessed by measuring fluorescence of liver metastases. In comparison with controls, FAM126A knockdown significantly inhibited metastasis in vivo (Fig. 4E). The nodes of the liver after H&E staining displayed liver metastasis (Fig. 4F, G).

### FAM126A interacted with ENO1 promoted PC growth and metastasis

**Fig. 5:**
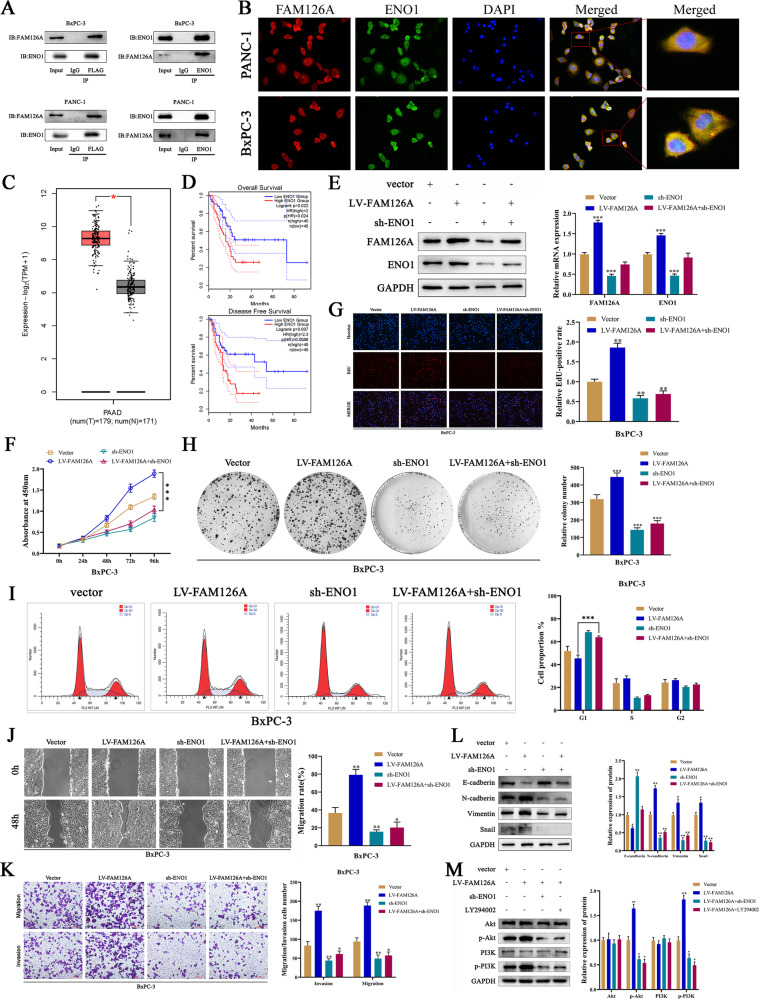
FAM126A overexpression suppresses the PI3K/AKT pathway and EMT through ENO1 downregulation. **A** Proteins interacting with Girdin were examined in PANC‑1 and BxPC‑3 cells by co‑IP. Cell lysates were analyzed by Western blotting. **B** In immunofluorescence-based co-localization assays, FAM126A interacted with ENO1 in the cytoplasm. **C** Relative ENO1 expression levels assessed in PC tissues according to the GEPIA2 database. **D** Kaplan–Meier curves for disease-free survival and overall survival in PC based on the GEPIA2 database. **E** ENO1 levels were elevated in cells overexpressing FAM126A and decreased after FAM126A knockdown, while FAM126A levels were decreased after ENO1 knockdown, as determined by qRT-PCR and immunoblot. GAPDH was utilized for normalization. **F**–**I** ENO1 silencing dampened FAM126A overexpression-induced BxPC-3 cell proliferation, as measured by CCK-8, EdU, clonogenic, cell-cycle distribution assays. **J**–**K** ENO1 silencing dampened FAM126A overexpression-induced BxPC-3 cell proliferation, as measured by wound healing and transwell assays. **L** Downregulation of ENO1 in BXPC-3-FAM126A cells reversed the upregulation of N-cadherin, Vimentin, and Snail by FAM126A, and the downregulation of E-cadherin. **M** Phosphorylated PI3K and Akt amounts in PANC‑1 and BxPC‑3 cells were examined by immunoblot. *P < 0.05 **P < 0.01, ***P < 0.001.

ENO1 has a critical role in tumor progression [27–29]. Co-immunoprecipitation combined with mass spectrometry analysis revealed FAM126A interacts with a large number of binding partners, including ENO1. Then, we confirmed the direct interaction between FAM126A and ENO1 in a range of cell lines by co-immunoprecipitation assays (Fig. 5A). Meanwhile, we performed immunofluorescence co-localization assays and found that FAM126A interacted with ENO1 in the cytoplasm (Fig. 5B). In addition, ENO1 mRNA levels in PC tissue samples were markedly increased compared with those of adjacent non-cancerous tissue samples in the GEPIA2 database (Fig. 5C). Moreover, PC cases showing elevated ENO1 expression had reduced OS and DFS rates in comparison with those lowly expressing ENO1 (Fig. 5D). Furthermore, immunoblot revealed ENO1 amounts were elevated in cells with FAM126A overexpression and decreased in FAM126A knock-downed cells, while FAM126A levels were decreased after ENO1 knockdown (Fig. 5E). Taken together, these data indicated that FAM126A interacts with ENO1 in the cytoplasm. A previous study indicated that ENO1 induces pathological EMT [30–32]. To assess whether FAM126A induces EMT via ENO1 regulation, stable ENO1-knockdown BxPC-3 FAM126A (BxPC-3-FAM126A-shENO1), shENO1, and control cells were generated. ENO1 level reduction was verified both in terms of gene and protein expressions (Fig. 5E). ENO1 silencing blunted FAM126A overexpression-dependent BxPC-3 cell proliferation and metastasis, as assessed by CCK-8, EdU, clonogenic, cell cycle, wound healing, and transwell assays (Fig. 5F–K). Furthermore, downregulation of ENO1 in BXPC-3-FAM126A cells reversed the upregulation of N-cadherin, Vimentin, and Snail by FAM126A, as well as E-cadherin downregulation (Fig. 5L). The above data indicate ENO1 plays a critical role in FAM126A-mediated EMT.

### ENO1 is regulated in FAM126A-mediated PI3K/AKT signaling

A previous study showed ENO1 contributes to tumor progression through PI3K/AKT signaling [33]. As a result, knockdown of ENO1 remarkably reduced phosphorylated PI3K and AKT amounts, while leaving total protein amounts intact. Then, FAM126A overexpression in BxPC-3 cells treated with LY294002 had similar effects on p-PI3K and p-AKT (Fig. 5M). The above findings indicate ENO1 may be an upstream effector of PI3K/AKT signaling in PC, and is essential for FAM126A-mediated PC progression.

### ENO1 knockdown inhibits FAM126A-promoted liver metastasis in mice

**Fig. 6:**
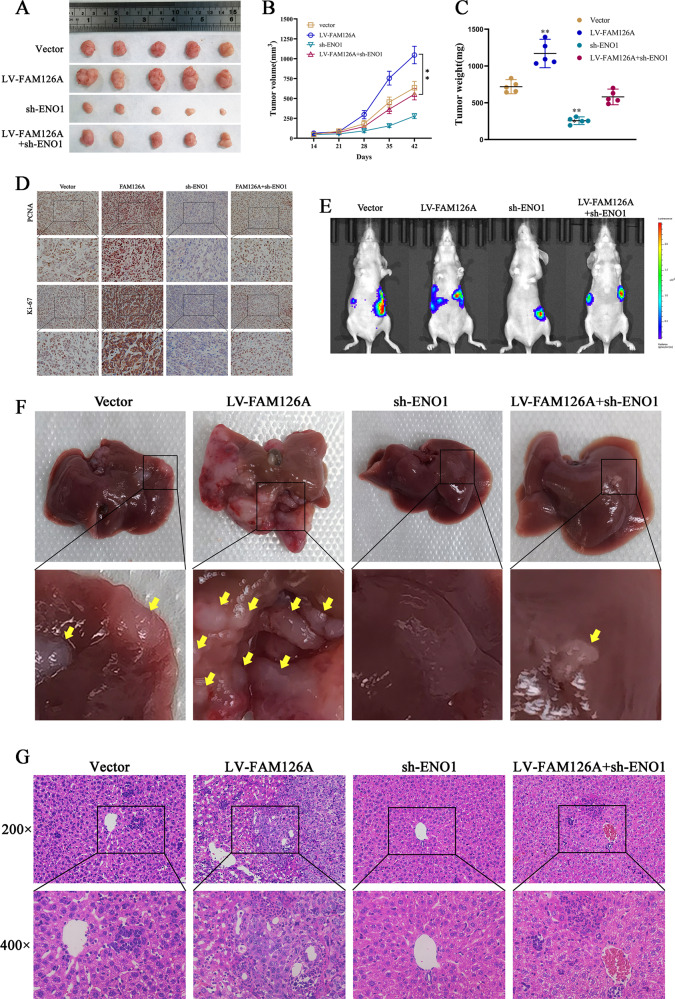
Effects of FAM126A and ENO1 on PC cell proliferation in vivo. **A** Vector, LV-FAM126A, sh-ENO1, and LV-FAM126A + sh-ENO1 cells were administered by the subcutaneous route to 4 mouse groups for xenograft model establishment. After 42 days, xenografts were extracted and imaged. **B** PC cell-xenograft tumor volumes were assessed weekly for 42 days. **C** Upon euthanasia, tumors were weighed. **D** IHC showing Ki67 and PCNA protein amounts in tumor samples. **E** Liver metastatic tumor model establishment was performed via injection of PC stable cell suspensions into the mouse spleen. **F** Representative images of liver specimens. Arrow, metastatic tumor. **G** H&E staining of liver metastatic nodules in various groups. *P < 0.05 **P < 0.01, ***P < 0.001.

To further clarify the roles of FAM126A and ENO1 in vivo, stable vector, FAM126A overexpression, LV-FAM126A + sh-ENO1, and shENO1 cell lines were established. Subsequently, a xenograft tumor model was constructed, and the growth of xenograft tumors was significantly inhibited by the downregulation of ENO1 in the LV-FAM126A group (Fig. 6A). Additionally, tumor weight was significantly higher in the LV-FAM126A group compared with the vector group. However, sh-ENO1 reversed the effect of LV-FAM126A (Fig. 6B, C). Immunohistochemistry and mRNA detection were performed on the tumor, and the results confirmed that the expression of FAM126A and ENO1 was consistent with the results verified before the tumogenesis experiment in nude mice (Fig. S1). IHC staining showed that ENO1 was knocked down, and the expression of Ki67 and PCNA was reduced in the LV-FAM126A group compared with the vector group (Fig. 6D). What’s more, the LV-FAM126A + sh-ENO1 group reversed all the effects observed in the LV-FAM126A group. Next, we established a liver metastatic tumor model via injection of PC stable cell suspension into the mouse spleen. After 4 weeks, the metastatic ability was assessed by measuring the fluorescence of liver metastases. In comparison with controls, FAM126A overexpression markedly induced metastasis in vivo, while knockdown of ENO1 reversed this phenotype (Fig. 6E). The nodes of the liver after H&E staining displayed liver metastasis (Fig. 6F). In H&E staining, the number of cancer cells in the sh-ENO1 group was reduced (Fig. 6G). These results fitted previously reported findings revealing ENO1 as an oncogene in cancer and showing FAM126A constitutes an oncogene by promoting the function of ENO1.

## Discussion

PC is highly invasive malignancy and approximately 85% of patients with PC were not surgically resectable at diagnosis and hence study of the underlying mechanisms of PC progress are critical [34, 35]. In the present study, we firstly investigated the association of FAM126A with PC tumorigenesis. After analysis of GEPIA2, GEO, and LOGpc database, we confirmed that FAM126A expression was elevated in PC compared to normal tissues. Moreover, the Kaplan-Meier survival curves demonstrated that overexpression of FAM126A predicted poor prognosis in PC, indicating the potential clinical value of FAM126A in PC.

Tumor progression is a multi-step, complex process, in which genetic changes are the very critical factors leading to the development of tumor [36, 37]. Family of sequence similarity, some of these families are associated with tumor development [11]. However, up to now, there has been no study on the biological function and related mechanism of FAM126A in cancer. In the current study, we observed that the expression level of FAM126A was significantly increased in PC. In vitro experiments on clone formation, CCK-8 assay, wound healing, and transwell have shown that overexpression of FAM126A increases the proliferation and metastasis in BxPC-3 cells, while FAM126A knockdown decreased the proliferation and metastasis of PANC-1 cells. Meanwhile, FAM126A knockdown also inhibited PC growth in vivo. Therefore, we hypothesize that FAM126A is an important factor in pancreatic cancer cell development.

Activation of EMT is thought to lead to cancer metastatic cascades [38, 39]. Previous studies have shown that EMT is associated with the metastasis of PC [40]. The relevant analysis of GEPIA2 manifested that FAM126A was direct correlated with Snail and Vimentin in PC tissues. Thus, we examined the expression of EMT-related transcription factors. We found that abnormal expression of FAM126A up-regulated N-cadherin, vimentin, and Snail, while down-regulated E-cadherin. In contrast, decreased expression of FAM126A led to upregulate of E-cadherin and down-regulate of N-cadherin, vimentin, and Snail. Morphological changes of BxPC-3 and PANC-1 also supported the above results. The results showed that FAM126A induces EMT in PC cells.

EMT is a reversible and conserved biological process, which is mainly regulated by PI3K/AKT signal and promotes the metastasis and malignant progression of various tumors [41, 42]. The PI3K/ AKT pathway is a signaling pathway associated with the development of many cancers, and the PI3K/ AKT pathway is abnormally activated during the development of PC [43, 44]. Studies have found that PI3K/ AKT pathway is a key regulator of pancreatic tumorigenesis and may become a therapy target for cancer [45, 46]. It has been reported that knockdown of ENO1 expression can inhibit the proliferation and metastasis of tumor cells by inhibiting the phosphorylation/activation of the PI3K/ AKT pathway [33].

It is acknowledged that ENO1 plays a significant role in tumor progression [47]. FAM126A may interact with ENO1, which was found by co-immunoprecipitation and immunofluorescence staining. Further studies on the roles of FAM126A and ENO1 in the PI3K/ AKT pathway can help to announce the potential molecular mechanism on the regulation of PC by FAM126A. As is shown in this study, overexpression of FAM126A increased p-AKT and p-PI3K levels in PC cell lines through the PI3K/ AKT pathway, and knockdown of ENO1 reversed this phenotype, similar to the effect of the PI3K inhibitor LY294002. ENO1 knockdown reversed the FAM126A-induced PI3K/ AKT pathway activation and enhanced proliferation and migration, and we demonstrated that ENO1 is necessary in the FAM126A-mediated activation of the PI3K/ AKT pathway.

Hence, we considered that FAM126A may control the transcription level of ENO1. Along with transcriptional alteration of ENO1 and activated PI3K/AKT signaling pathway, the expression of Cyclin D1, Cyclin E1, CDK2, CDK4, and EMT markers were altered, which then lead to a transformation in biological behavior of PC cells. Yet, the more specific mechanisms still need to be further explored in the future. In summary, this study confirmed that FAM126A has a crucial effect in the proliferation and distant metastasis of PC cells. FAM126A activates PI3K/AKT signaling pathway through altering the expression level of ENO1. Activation of PI3K/AKT signaling pathway induces EMT and promotes cancer metastasis.

## Materials and methods

### Data source and bioinformatics analysis

We first searched the Gene Expression Omnibus (GEO) database for data sets containing pancreatic cancer and normal tissue, excluding chemotherapy or radiation treatment. We obtained three data sets (GSE15471, GSE11838, and GSE16515) containing 113 cancer and 13 nonmalignant specimens, 13 cancer and 9 nonmalignant specimens, and 39 pairs of cancer and normal specimens, respectively. GraphPad Prism 6 was utilized for analyzing data from The Cancer Genome Atlas (TCGA, https://cancergenome.nih.gov). By Gene Expression Profiling Interactive Analysis 2 (GEPIA2, http://gepia2.cancer-pku.cn/#general), a great tool for analyzing gene expression in cancer and noncancerous specimens based on TCGA and the GTEx [48], FAM126A amounts in pancreatic malignant and noncancerous tissues were assessed. A boxplot was generated for visualizing the association. The Long-term Outcome and Gene Expression Profiling Database of pan-cancers (LOGpc, http://bioinfo.henu.edu.cn/DatabaseList.jsp) represents a webserver containing multiple datasets for survival analysis, with specimens mostly obtained from TCGA and GEO cohorts [49]. Then, overall survival in PC was examined based on FAM126A expression.

### Human tissue samples

Forty-nine (49) surgical resected primary PDAC tissues and paired para cancer pancreatic tissues, as well as non-cancerous pancreatic tissues (2 cm away from tumor edge), were obtained from the Affiliated Hospital of Guizhou Medical University. To avoid any changes in tumor marker determination due to the effect of the treatment, patients who had received preoperative chemotherapy, radiotherapy, or biotherapy were excluded from the study. Signed informed consent was provided by each patient. The PC tissue microarray was provided by Shanghai Outdo Biotech (China). The present study had approval from the Human Research Ethics Committee at the Affiliated Hospital of Guizhou Medical University.

### Chemicals and cells

AsPC-1, BxPC-3, Capan-2, Mia PaCa-2, PANC-1, and SW1990 cells were provided by the American Type Culture Collection (ATCC; USA), after authentication by short tandem repeat typing and testing for mycoplasma contamination. The Capan-2, Mia PaCa-2, PANC-1, and SW1990 cell lines underwent culture in DMEM (Gibco, USA), and AsPC-1 and BxPC-3 cell culture utilized RPMI 1640 (Gibco, USA) containing 10% fetal bovine serum (Gibco), 100 U/mL penicillin, 100 µg/mL streptomycin and 2 mM L-Glutamine, in a humid environment with 5% CO_2_ at 37 °C.

### RNA Preparation

Total RNA was extracted from the cell lines and pancreatic tissues using Trizol reagent (Invitrogen, CA, USA). The concentration and quality of isolated RNA were detected by the spectrophotometer (NanoDrop™ One/OneC, Thermo Scientific). Complementary DNA (cDNA) was generated for mRNA analysis using oligo-dT primers and PrimeScript RT reagent kit (Takara, Beijing, China).

### Quantitative real-time PCR

Amplification of the generated cDNA was detected using TB Green Premix ExTaq (Takara) on CFX96^TM^ Real-Time system (Bio-Rad, CA, USA). Primer pairs were: FAM126A, sense 5′-CAGAGAGCCCAAAGTGAGA-3, and antisense 3′-AACAGCAGATGACGGGTTA-5′; ENO1, sense 5′-GCCGGCTTTACGTTCACCTC-3′and antisense 5′-GTTGAAGCACCACTGGGCAC-3′; GAPDH (glyceraldehyde-3-phosphate dehydrogenase), sense 5′-CCACAGTCCATGCCATCACTG-3′and antisense 5′-GTCAGGTCCACCACTGACACG-3′. GAPDH was used as an internal control, and the differences of gene expression were calculated by 2^−ΔΔCt^ method.

### Western blot analysis

Tumor tissues and cells were washed with PBS and underwent lysis with RIPA buffer (Pierce) supplemented with protease inhibitors (Boster Biological Technology, China). Protein quantitation utilized the BCA assay kit (Beyotime Biotechnology). Equal amounts of total protein were resolved by SDS-PAGE and underwent transfer onto polyvinylidene fluoride membranes (Millipore, USA). Western blot membranes underwent overnight incubation at 4 °C with anti-FAM126A (1:1000; Sigma, #84668), anti-ENO1 (1:1000; Proteintech, #11204), anti-GHPDH (1:1000; Proteintech, #60004), anti‑E‑cadherin (1:1000; Proteintech, #20874), anti-N-cadherin (1:1000; Proteintech, #22018), anti‑vimentin (1:1,000; Proteintech, #10366), anti-snail (1:1000; Proteintech, #13099), anti-cyclin D1 (1:1000; Cell Signaling Technology [CST], #55506), anti-cyclin E1 (1:1000; CST, #4136), anti-CDK 2 (1:1000; CST, #2561), anti-CDK4 (1:1,000; CST, #12790), anti-AKT (1:1000; CST, #4691), anti-p-AKT (1:2000; CST, #4060), anti-PI3K (1:1000; CST, #4249), anti-p-PI3K (1:1000; CST, #17366), anti-Ki-67(1:800; CST, #9449) and anti-PCNA (1:1000; CST, #2561) primary antibodies. This was followed by incubation with HRP-linked anti-rabbit IgG (Boster, #BA1041). Enhanced chemiluminescence reagent (Proteintech, #7003) was used to detect immunoreactive signals.

### Lentivirus infection

Lentiviral constructs carrying a FAM126A overexpression vector or expressing FAM126A encoding short hairpin RNA (shRNA), negative control, and shRNA targeting ENO1 were provided by GeneChem (China). PANC-1 and BxPC-3 cells underwent a 48 h transfection, with immunoblot and qRT-PCR verification. Three shRNA targeting the FAM126A gene were synthesized, and two with highest efficacy in downregulating FAM126A based on qRT-PCR were further assessed.

### Cell viability assay

Cells (5000/well in a 96-well plate) were incubated for 0, 24, 48, 72, and 96 h, and tested with Cell Counting Kit-8 (Dojindo, Japan). Absorbance was read at 450 nm.

### Colony-formation assay

PC BxPC-3 and PANC-1 cells (1000/well in six-well plates) underwent a 14-day culture, formalin (4%) fixation and crystal violet (0.25%) staining. Colonies counting and imaging were then performed.

### Flow cytometry

PC BxPC-3 and PANC-1 cell seeding into six-well plates was followed by incubation for 24 h. After harvest and PBS washes, incubation was carried out with DNA staining and permeabilization solutions (Cell Cycle Staining Kit, MULTI SCIENCES, China) for 30 min shielded from light at room temperature. Summit 5.2 (Beckman Coulter, USA) was utilized for analysis.

### Wound healing assay

When the confluence of cells reached 80–90% in a six-well plate, the tip of a 200 µl pipette was used to scratch the monolayer. This was followed by a 2–3-day incubation in media with no FBS. The remaining gaps were examined at various times.

### Transwell assays

Cell migration assay was carried out as follows. In a Transwell apparatus (CoStar, USA), 1 × 10^4^ cells were plated on fibronectin-coated polycarbonate membrane inserts in 200 μL DMEM without FBS. In the lower chamber, 800 μl DMEM containing 10% FBS was supplemented. Following an 8 h incubation in a 5% CO_2_ incubator at 37 °C, the insert underwent PBS washes, and cells on its top surface were removed with a cotton swab. Then, cells attached to the lower surface underwent methanol fixation, Giemsa staining, and counting by microscopy. The cell invasion assay was similarly performed, with the Transwell membrane precoated with Matrigel (R&D Systems, USA) at 24 μg/μl. All trials were repeated independently at least 3 times.

### Co-immunoprecipitation

Following a 48 h transfection, the cells were lysed with a chilled buffer supplemented with protease inhibitors for 30 min. Then, centrifugation was carried out at 14,000 × *g* and 4 °C for 10 min. Magnetic beads conjugated to respective antibodies were supplemented to supernatants and slowly shaken overnight at 4 °C. Coupled antigen-antibody beads. After immunoprecipitation, the magnetic bead-coupled complex underwent washes with the buffer solution. Then, 2× loading buffer was added and boiled for protein sample preparation. Finally, the interacting protein was identified by Western blotting.

### Mouse xenograft model

All mouse experiments were approved by the Animal Care Welfare Committee of Guizhou Medical University. To assess the impact of FAM126A silencing in vivo, six to seven-week old female BALB/c nude mice (20 g) were randomly divided into three groups with five in each group. No blinding was performed and no statistical methods were used to pre-determine sample sizes. PANC-1 cells (sh-NC, sh-FAM126A#1, and sh-FAM126A#3 groups) were stably transfected with the firefly luciferase gene.

To further clarify the roles of FAM126A and ENO1 in vivo, six to seven-week old female BALB/c nude mice (20 g) were randomly divided into four groups with five in each group. PANC-1 cells (Vector, FAM126A overexpression, sh-ENO1, and FAM126A overexpression+sh-ENO1 groups) were stably transfected with the firefly luciferase gene.

The indicated cells were inoculated by the subcutaneous route (2 × 10^6^/mouse) into the right axilla of nude mice. The IVIS imaging system was used to detect fluorescence in vivo. Tumor volumes were derived as (Length × Width^2^)/2, based on measurements with a Vernier caliper. Tumors were sectioned or lysed for further analysis.

### Immunohistochemistry

The expression of PCNA and Ki-67 proteins in PANC-1 xenografts were detected by IHC. Fresh tumors were fixed overnight at 4 °C, paraffin-embedded, and cut into 5 μm sections. After incubation with antibodies (overnight at 4 °C) the specimens were treated with the AEC chromogenic substrate, followed by hematoxylin counterstaining and microscopy.

### Statistical analysis

Continuous data are mean ± SD, and were compared by two-tailed Student’s t-test and one-way ANOVA for group pairs and multiple groups, respectively. GraphPad Prism 8.0 was utilized for data analysis. ImageJ V1.46 was used for image assessment. *P* < 0.05 indicated statistical significance.

## Supplementary information

Original Data File

All of the co-authors’ email responses

Supplemental Information

## Data Availability

The original contributions presented in the study are included in the article/Supplementary Material; further inquiries can be directed to the corresponding authors.
