# Supplementary figures and images for: FAM126A interacted with ENO1 mediates proliferation and metastasis in pancreatic cancer via PI3K/AKT signaling pathway
Source: Cell Death Discov. 2022 May 5;8:248. doi: 10.1038/s41420-022-01047-9 (PMC9072533; doi:10.1038/s41420-022-01047-9)

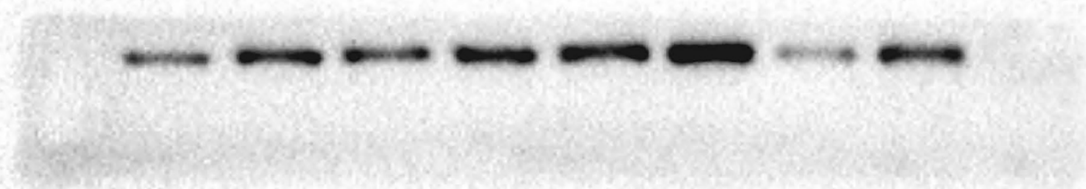

-----

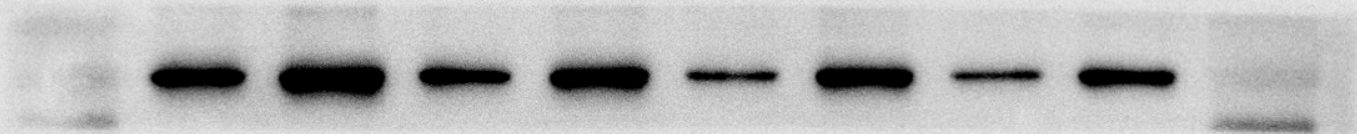

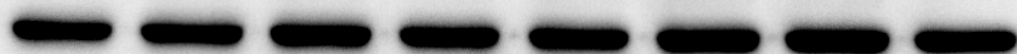

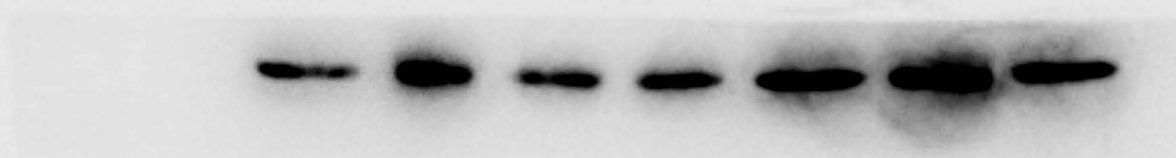

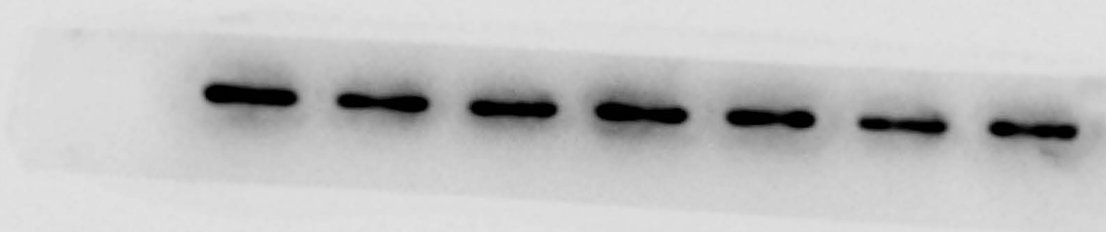



--

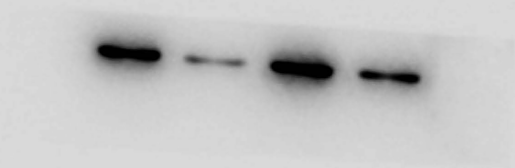

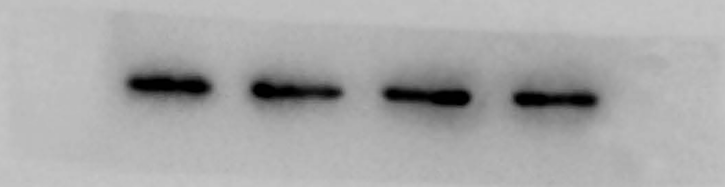

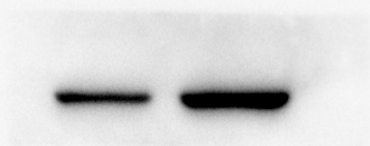

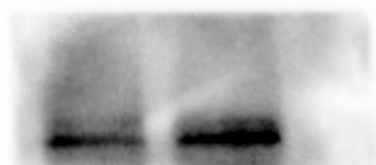

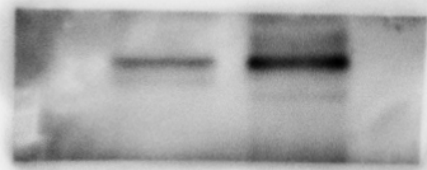

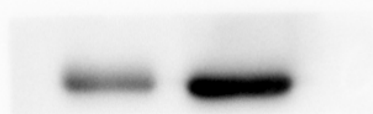

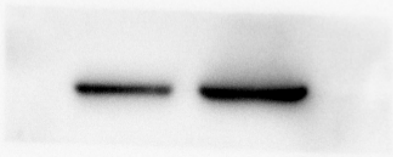

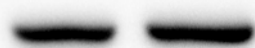

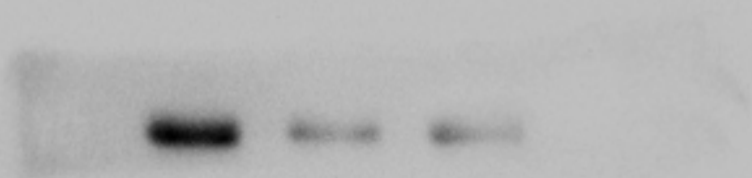

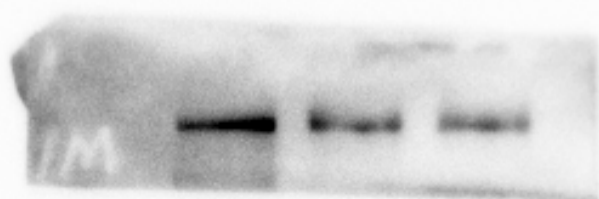

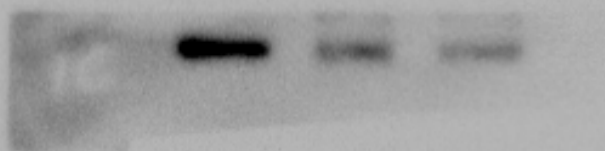

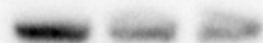

— — —

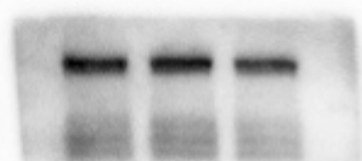

— —

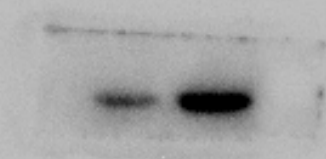

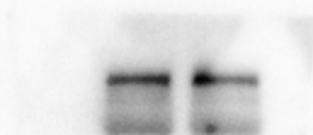

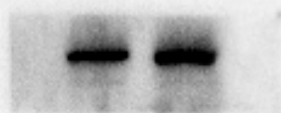

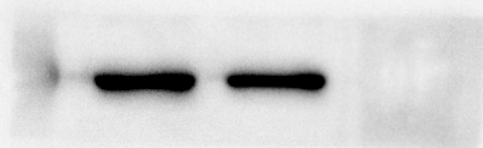

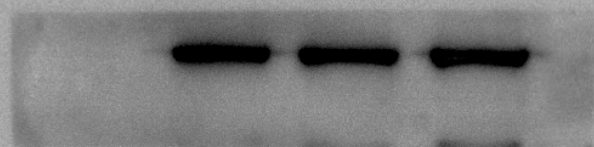

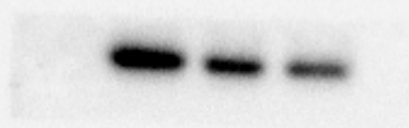

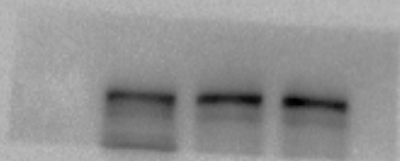

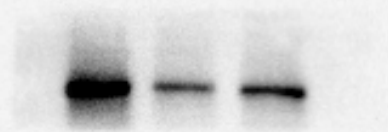

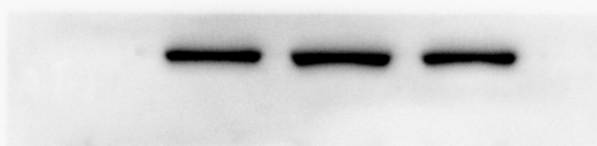

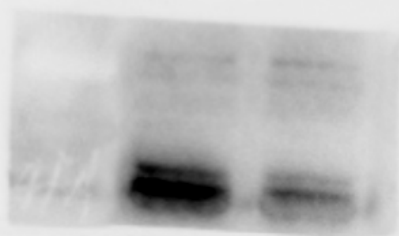

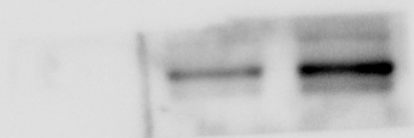

— —

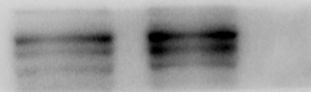

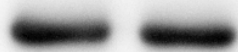

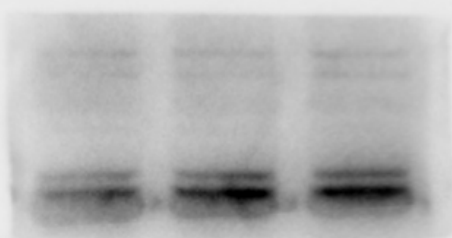

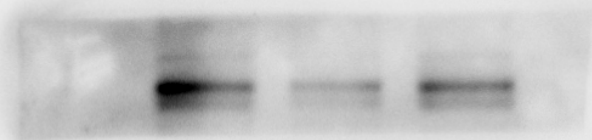

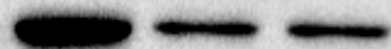

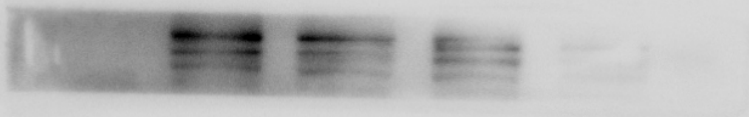

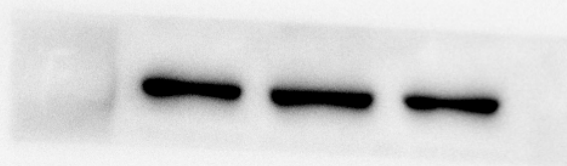

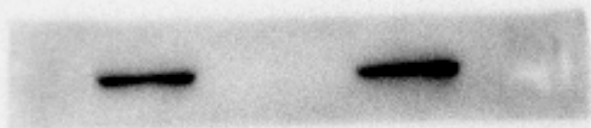

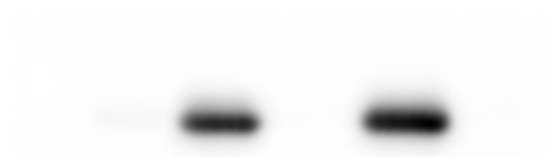

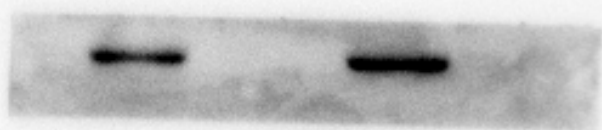

— —

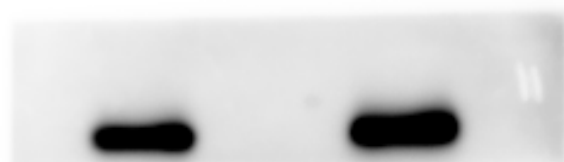

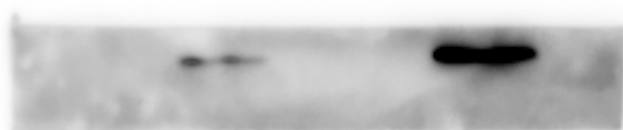

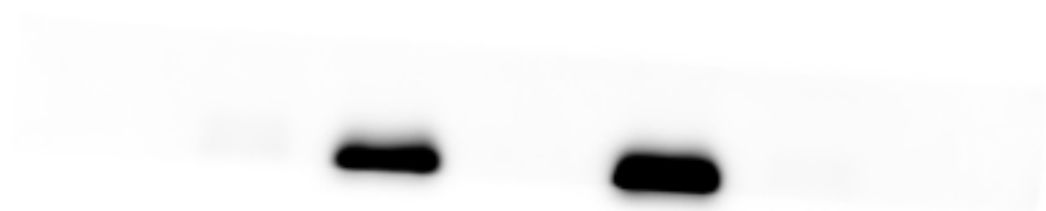

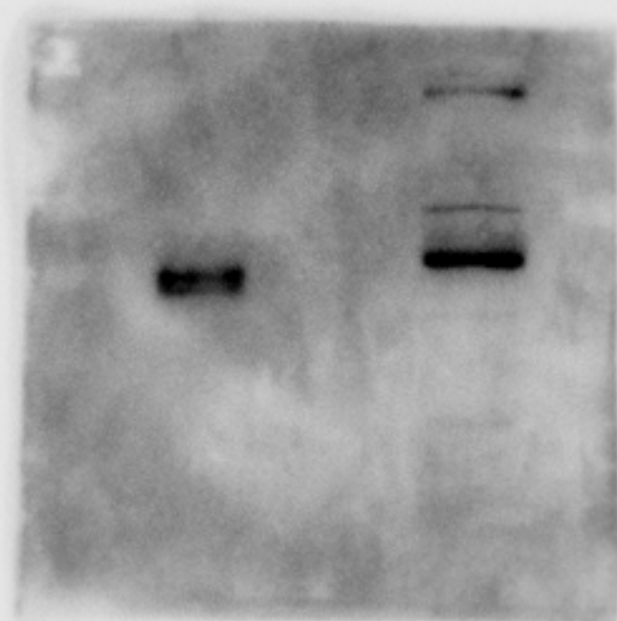

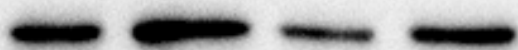

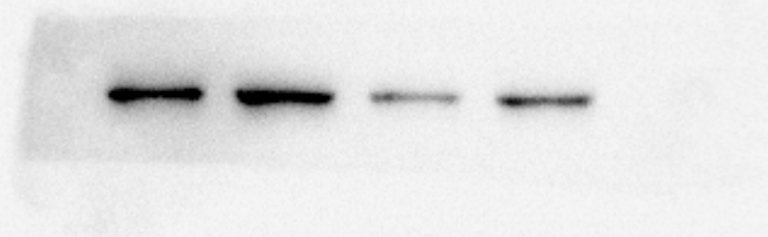

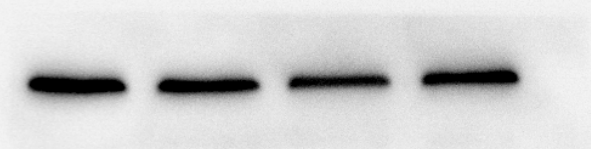

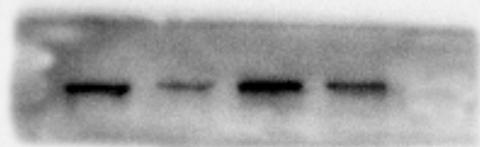

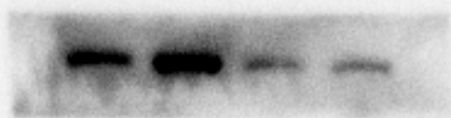

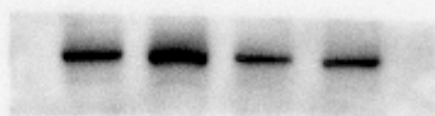

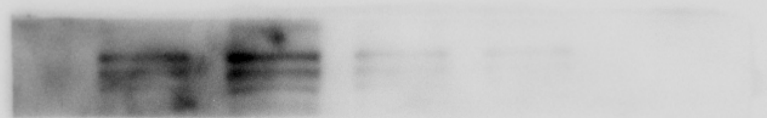

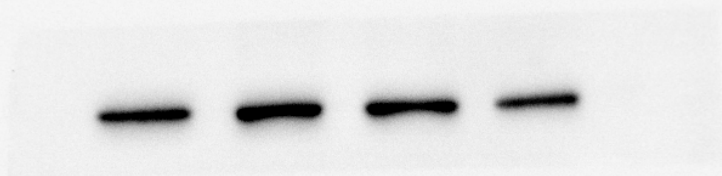

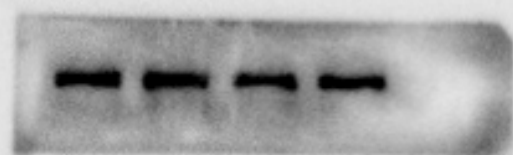

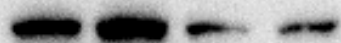

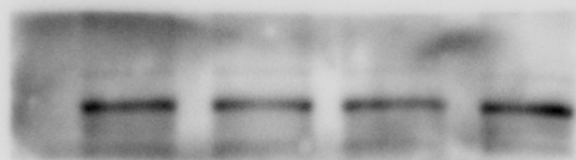

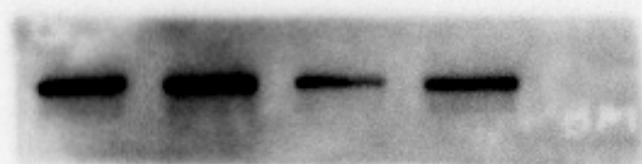

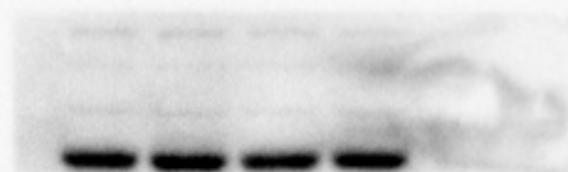

Supplement: Supplementary file 1 — Original Data File [file 41420_2022_1047_MOESM1_ESM.pdf]
