# Supplementary material for: FAM126A interacted with ENO1 mediates proliferation and metastasis in pancreatic cancer via PI3K/AKT signaling pathway
Source: Cell Death Discov. 2022 May 5;8:248. doi: 10.1038/s41420-022-01047-9 (PMC9072533; doi:10.1038/s41420-022-01047-9)
Supplement: Supplementary file 3 — Supplemental Information [file 41420_2022_1047_MOESM3_ESM.docx]

Supplemental Information

**Title: FAM126A Interacted with ENO1 Mediates Proliferation and Metastasis in Pancreatic Cancer via PI3K/AKT Signaling Pathway**

Yongning Li^1,2#^, Ying Li^1#^, Jun Luo^1,2#^, Peng Liu^1,2^, Songbai Liu^1^, Yaozhen Pan^1,3*^

^1^College of Clinical Medicine, Guizhou Medical University, Guiyang, Guizhou, China

^2^ Department of Hepatobiliary Surgery, The Affiliated Hospital of Guizhou Medical University, Guiyang, Guizhou, China

^3^Department of Hepatobiliary Surgery, The Affiliated Cancer Hospital of Guizhou Medical University, Guiyang, Guizhou, China

**Correspondence:** Yaozhen Pan，

**Address:** Department of Hepatobiliary Surgery, The Affiliated Cancer Hospital of Guizhou Medical University, Guiyang, Guizhou, China

**Email:** [panyaozhen@gmc.edu.cn](mailto:panyaozhen@gmc.edu.cn)

**Short title:** FAM126A acts oncogene in pancreatic cancer.

**#These authors contributed equally to this work：**Yongning Li, Ying Li, Jun Luo

**Supplementary Figure 1.**


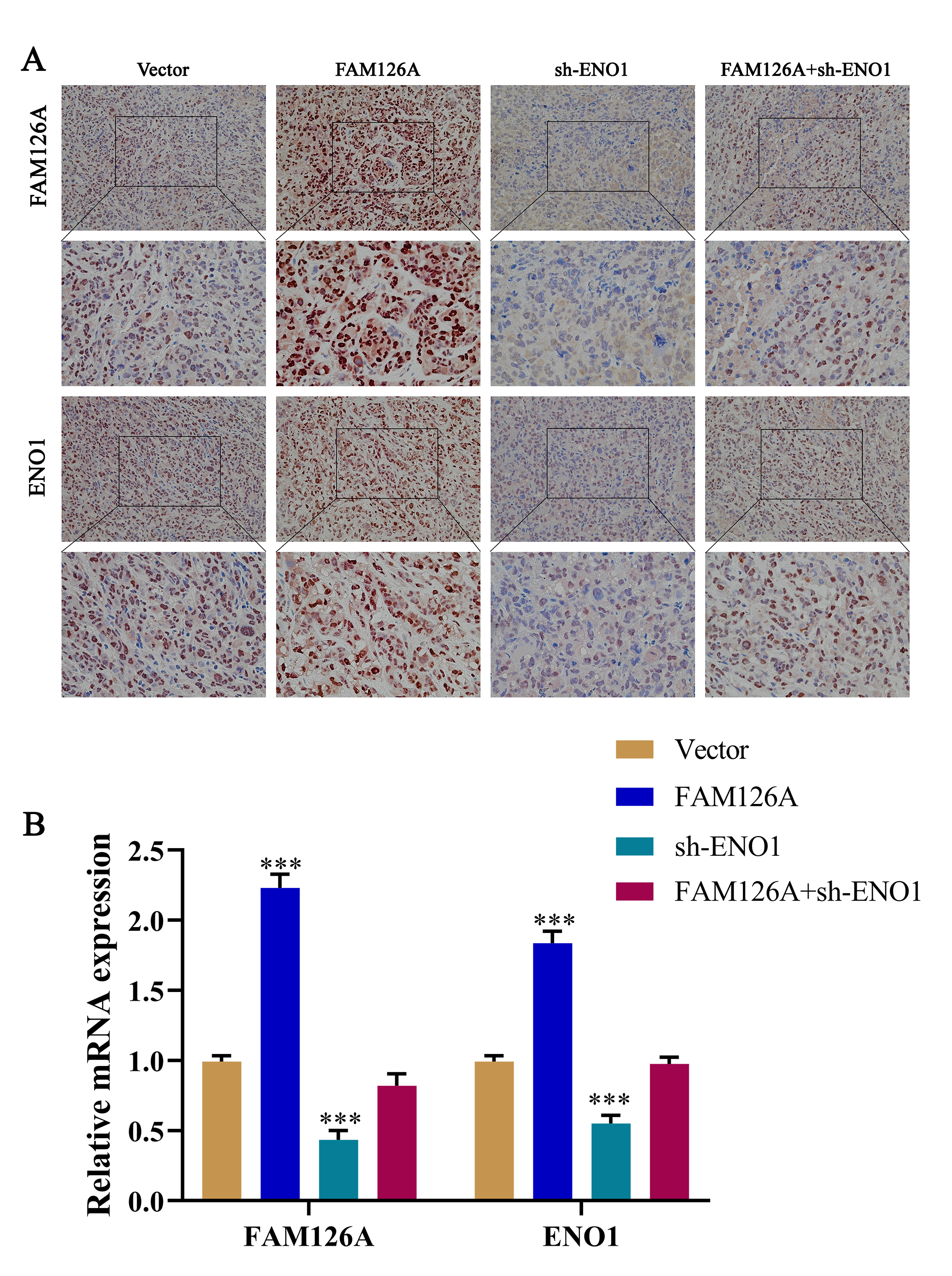


**Figure S1. The expression levels of FAM126A and ENO1 in nude mouse tumors.** **A.** The expression levels of FAM126A and ENO1 in nude mouse tumors were detected by immunohistochemistry. **B.** The expression levels of FAM126A and ENO1 in nude mouse tumors were detected by QRT-PCR.
