# Supplementary material for: FAM126A interacted with ENO1 mediates proliferation and metastasis in pancreatic cancer via PI3K/AKT signaling pathway
Source: Cell Death Discov. 2022 May 5;8:248. doi: 10.1038/s41420-022-01047-9 (PMC9072533; doi:10.1038/s41420-022-01047-9)
Supplement: Supplementary file 2 — All of the co-authors’ email responses [file 41420_2022_1047_MOESM2_ESM.pdf]

Dear Dr Pan,

About：Manuscript Number: CDDISCOVERY-22-3681R1
Title: FAM126A Interacted with ENO1 Mediates Proliferation and Metastasis in Pancreatic Cancer via PI3K/AKT Signaling Pathway

Yes, I agree to add Xueqin Fu to the author list.

Yours sincerely,

Yongning Li
Guizhou Medical University

-----Original Messages-----
From:"潘耀振" <panyaozhen@gmc.edu.cn>
Sent Time:2022-04-26 19:41:28 (Tuesday)
To: yongninglee@gmc.edu.cn
Cc:
Subject: Change the original author list
Dear Dr Li,

About：Manuscript Number: CDDISCOVERY-22-3681R1
Title: FAM126A Interacted with ENO1 Mediates Proliferation and Metastasis in Pancreatic Cancer via PI3K/AKT Signaling Pathway

Author Xueqin Fu will be added to the list of authors for her contributions to thesis writing and native language polishing.
Now change the original author list: Yaozhen Pan, Yongning Li, Ying Li, Jun Luo, Peng Liu, and Songbai Liu **to** Yaozhen Pan, Yongning Li, Ying Li, Jun Luo, **Xueqin Fu,** Peng Liu, and Songbai Liu. Do you agree?

Yours sincerely,

Yaozhen Pan
Guizhou Medical University
panyaozhen@gmc.edu.cn

Lock Screen Logout Search Messages

New

Inbox
Todo
Draft
Sent
My Folders

Inbox | Re: Chang... ×

Reply | Reply All | Forward | Move | Mark | More | Delete

Re: Change the original author list

李永宁 send to 潘耀振

2022-04-26 19:51:19

Dear Dr Pan,

About：Manuscript Number: CDDISCOVERY-22-3681R1
Title: FAM126A Interacted with ENO1 Mediates Proliferation and Metastasis in Pancreatic Cancer via PI3K/AKT Signaling Pathway

Yes, I agree to add Xueqin Fu to the author list.

Yours sincerely,

Yongning Li

Guizhou Medical University

-----Original Messages-----
From:"潘耀振" <panyaozhen@gmc.edu.cn>
Sent Time:2022-04-26 19:41:28 (Tuesday)
To: yongninglee@gmc.edu.cn
Cc:
Subject: Change the original author list

Dear Dr Li,

About：Manuscript Number: CDDISCOVERY-22-3681R1
Title: FAM126A Interacted with ENO1 Mediates Proliferation and Metastasis in Pancreatic Cancer via PI3K/AKT Signaling Pathway

Author Xueqin Fu will be added to the list of authors for her contributions to thesis writing and native language polishing.

Now change the original author list: Yaozhen Pan, Yongning Li, Ying Li, Jun Luo, Peng Liu, and Songbai Liu **to** Yaozhen Pan, Yongning Li, Ying Li, Jun Luo, **Xueqin Fu,** Peng Liu, and Songbai Liu. Do you agree?

Yours sincerely,

Yaozhen Pan

Guizhou Medical University

panyaozhen@gmc.edu.cn

Reply to all

Dear Dr Pan,

About：Manuscript Number: CDDISCOVERY-22-3681R1

Title: FAM126A Interacted with ENO1 Mediates Proliferation and Metastasis in Pancreatic Cancer via PI3K/AKT Signaling Pathway.
Yes, I agree to add Xueqin Fu to the author list.
Yours sincerely,
YingLi
Guizhou Medical University

yinglee1996

邮箱：yinglee1996@yeah.net

签名由 网易邮箱大师 定制

On 04/26/2022 19:58, 潘耀振 wrote:

Dear Ms Li,

About：Manuscript Number: CDDISCOVERY-22-3681R1
Title: FAM126A Interacted with ENO1 Mediates Proliferation and Metastasis in Pancreatic Cancer via PI3K/AKT Signaling Pathway

Author Xueqin Fu will be added to the list of authors for her contributions to thesis writing and native language polishing.

Now change the original author list: Yaozhen Pan, Yongning Li, Ying Li, Jun Luo, Peng Liu, and Songbai Liu **to** Yaozhen Pan, Yongning Li, Ying Li, Jun Luo, **Xueqin Fu,** Peng Liu, and Songbai Liu. Do you agree?

Yours sincerely,

Yaozhen Pan
Guizhou Medical University
panyaozhen@gmc.edu.cn

Lock Screen Logout Search Messages

New

Inbox
Todo
Draft
Sent
My Folders

Inbox | 回复：Cha... | Re: Chang...

Reply | Reply All | Forward | Move | Mark | More | Delete

Re: Change the original author list

yinglee1996@yeah.net send to 潘耀振

2022-04-26 20:06:21

Dear Dr Pan,
About：Manuscript Number: CDDISCOVERY-22-3681R1
Title: FAM126A Interacted with ENO1 Mediates Proliferation and Metastasis in Pancreatic Cancer via PI3K/AKT Signaling Pathway.
Yes, I agree to add Xueqin Fu to the author list.
Yours sincerely,
YingLi
Guizhou Medical University

yinglee1996

邮箱：yinglee1996@yeah.net

签名由 网易邮箱大师 定制

On 04/26/2022 19:58, 潘耀振 wrote:

Dear Ms Li,

About: Manuscript Number: CDDISCOVERY-22-3681R1
Title: FAM126A Interacted with ENO1 Mediates Proliferation and Metastasis in Pancreatic Cancer via PI3K/AKT Signaling Pathway

Author Xueqin Fu will be added to the list of authors for her contributions to thesis writing and native language polishing.

Now change the original author list: Yaozhen Pan, Yongning Li, Ying Li, Jun Luo, Peng Liu, and Songbai Liu **to** Yaozhen Pan, Yongning Li, Ying Li, Jun Luo, **Xueqin Fu,** Peng Liu, and Songbai Liu. Do you agree?

Yours sincerely,

Yaozhen Pan

Guizhou Medical University

panyaozhen@gmc.edu.cn

Reply to all

Yes, I agree this new author list.

Luo Jun

焰火
260669665@qq.com

------------------ 原始邮件 ------------------
发件人: "潘耀振" <panyaozhen@gmc.edu.cn>;
发送时间: 2022 年 4 月 26 日(星期二) 晚上 7:59
收件人: "焰火"<260669665@qq.com>;
主题: Change the original author list

Dear Dr Luo,

About: Manuscript Number: CDDISCOVERY-22-3681R1
Title: FAM126A Interacted with ENO1 Mediates Proliferation and Metastasis in Pancreatic Cancer via PI3K/AKT Signaling Pathway

Author Xueqin Fu will be added to the list of authors for her contributions to thesis writing and native language polishing.

Now change the original author list: Yaozhen Pan, Yongning Li, Ying Li, Jun Luo, Peng Liu, and Songbai Liu to Yaozhen Pan, Yongning Li, Ying Li, Jun Luo, Xueqin Fu, Peng Liu, and Songbai Liu. Do you agree?

Yours sincerely,

Yaozhen Pan
Guizhou Medical University
panyaozhen@gmc.edu.cn

电子邮箱

Lock Screen Logout Search Messages

New

Inbox

Todo

Draft

Sent

My Folders

Inbox 回复：Cha...

Reply Reply All Forward Move Mark More Delete

回复：Change the original author list

焰火 send to 潘耀振

2022-04-26 21:43:12

Yes, I agree this new author list.

Luo Jun

焰火

260669665@qq.com

------------------ 原始邮件 ------------------

发件人: "潘耀振" <panyaozhen@gmc.edu.cn>;
发送时间: 2022年4月26日(星期二) 晚上7:59
收件人: "焰火"<260669665@qq.com>;
主题: Change the original author list

Dear Dr Luo,

About: Manuscript Number: CDDISCOVERY-22-3681R1
Title: FAM126A Interacted with ENO1 Mediates Proliferation and Metastasis in Pancreatic Cancer via PI3K/AKT Signaling Pathway

Author Xueqin Fu will be added to the list of authors for her contributions to thesis writing and native language polishing.

Now change the original author list: Yaozhen Pan, Yongning Li, Ying Li, Jun Luo, Peng Liu, and Songbai Liu **to** Yaozhen Pan, Yongning Li, Ying Li, Jun Luo, **Xueqin Fu,** Peng Liu, and Songbai Liu. Do you agree?

Yours sincerely,

Yaozhen Pan

Guizhou Medical University

panyaozhen@gmc.edu.cn

Reply to all

Dear Dr Pan,

About：Manuscript Number: CDDISCOVERY-22-3681R1

Title: FAM126A Interacted with ENO1 Mediates Proliferation and Metastasis in Pancreatic Cancer via PI3K/AKT Signaling Pathway

Yes, I agree to add Xueqin Fu to the author list.

Yours sincerely,

Xueqin Fu

Guizhou Provincial People's Hospital

------------------ 原始邮件 ------------------

发件人: "潘耀振" <panyaozhen@gmc.edu.cn>;

发送时间: 2022 年 4 月 26 日(星期二) 晚上 8:03

收件人: "506012287"<506012287@qq.com>;

主题: Change the original author list

Dear Ms Fu,

About：Manuscript Number: CDDISCOVERY-22-3681R1

Title: FAM126A Interacted with ENO1 Mediates Proliferation and Metastasis in Pancreatic Cancer via PI3K/AKT Signaling Pathway

You will be added to the list of authors for your contributions to thesis writing and native language polishing.

Now change the original author list: Yaozhen Pan, Yongning Li, Ying Li, Jun Luo, Peng Liu, and Songbai Liu to Yaozhen Pan, Yongning Li, Ying Li, Jun Luo, Xueqin Fu, Peng Liu, and Songbai Liu. Do you agree?

Yours sincerely,

Yaozhen Pan

Guizhou Medical University

panyaozhen@gmc.edu.cn

电子邮箱

Lock Screen Logout Search Messages

New

Inbox

Todo

Draft

Sent

My Folders

Inbox | 回复：Cha... | Re: Chang...

Reply | Reply All | Forward | Move | Mark | More | Delete

回复：Change the original author list

506012287 send to 潘耀振

2022-04-26 20:11:03

Dear Dr Pan,

About：Manuscript Number: CDDISCOVERY-22-3681R1

Title: FAM126A Interacted with ENO1 Mediates Proliferation and Metastasis in Pancreatic Cancer via PI3K/AKT Signaling Pathway

Yes, I agree to add Xueqin Fu to the author list.

Yours sincerely,

Xueqin Fu

Guizhou Provincial People's Hospital

------------------ 原始邮件 ------------------

发件人： "潘耀振" <panyaozhen@gmc.edu.cn>;
发送时间： 2022年4月26日(星期二) 晚上8:03
收件人： "506012287"<506012287@qq.com>;
主题： Change the original author list

Dear Ms Fu,

About: Manuscript Number: CDDISCOVERY-22-3681R1
Title: FAM126A Interacted with ENO1 Mediates Proliferation and Metastasis in Pancreatic Cancer via PI3K/AKT Signaling Pathway

You will be added to the list of authors for your contributions to thesis writing and native language polishing.

Now change the original author list: Yaozhen Pan, Yongning Li, Ying Li, Jun Luo, Peng Liu, and Songbai Liu **to** Yaozhen Pan, Yongning Li, Ying Li, Jun Luo, **Xueqin Fu,** Peng Liu, and Songbai Liu. Do you agree?

Yours sincerely,

Yaozhen Pan

Guizhou Medical University

panyaozhen@gmc.edu.cn

Reply to all

Dear Dr Pan

About：Manuscript Number: CDDISCOVERY-22-3681R1Title: FAM126A Interacted with ENO1 Mediates Proliferation and Metastasis in Pancreatic Cancer via PI3K/AKT Signaling PathwayYes, I agree to add Xueqin Fu to the author list.Yours sincerely

Peng Liu

Guizhou Medical University

-------- 原始邮件 --------

发件人： 潘耀振 <panyaozhen@gmc.edu.cn>

日期： 2022 年 4 月 26 日周二 晚上 8:04

收件人： liupeng5061@126.com

主 题： Change the original author list

Dear Mr Liu,

About：Manuscript Number: CDDISCOVERY-22-3681R1

Title: FAM126A Interacted with ENO1 Mediates Proliferation and Metastasis in Pancreatic Cancer via PI3K/AKT Signaling Pathway

Author Xueqin Fu will be added to the list of authors for her contributions to thesis writing and native language polishing.

Now change the original author list: Yaozhen Pan, Yongning Li, Ying Li, Jun Luo, Peng Liu, and Songbai Liu to Yaozhen Pan, Yongning Li, Ying Li, Jun Luo, Xueqin Fu, Peng Liu, and Songbai Liu. Do you agree?

Yours sincerely,

Yaozhen Pan
Guizhou Medical University
panyaozhen@gmc.edu.cn

Lock Screen Logout Search Messages

New

Inbox
Todo
Draft
Sent
My Folders

Inbox | 回复：Cha... | 回复：Cha...

Reply | Reply All | Forward | Move | Mark | More | Delete

回复：Change the original author list

liupeng5061@126.com send to 潘耀振

2022-04-26 20:45:45

Dear Dr Pan
About：Manuscript Number：CDDISCOVERY-22-3681R1Title：FAM126A Interacted with ENO1 Mediates Proliferation and Metastasis in Pancreatic Cancer via PI3K/AKT Signaling PathwayYes，I agree to add Xueqin Fu to the author list.Yours sincerely
Peng Liu
Guizhou Medical University

发自我的手机

-------- 原始邮件 --------
发件人： 潘耀振 <panyaozhen@gmc.edu.cn>
日期： 2022年4月26日周二 晚上8:04
收件人： liupeng5061@126.com
主 题： Change the original author list

Dear Mr Liu,

About：Manuscript Number：CDDISCOVERY-22-3681R1
Title：FAM126A Interacted with ENO1 Mediates Proliferation and Metastasis in Pancreatic Cancer via PI3K/AKT Signaling Pathway

Author Xueqin Fu will be added to the list of authors for her contributions to thesis writing and native language polishing.

Now change the original author list：Yaozhen Pan，Yongning Li，Ying Li，Jun Luo，Peng Liu，and Songbai Liu **to** Yaozhen Pan，Yongning Li，Ying Li，Jun Luo，**Xueqin Fu，** Peng Liu，and Songbai Liu. Do you agree?

Yours sincerely,

Yaozhen Pan

Guizhou Medical University

panyaozhen@gmc.edu.cn

Reply to all

Dear Dr Pan,

About：Manuscript Number: CDDISCOVERY-22-3681R1
Title: FAM126A Interacted with ENO1 Mediates Proliferation and Metastasis in Pancreatic Cancer via PI3K/AKT Signaling Pathway

Yes, I agree to add Xueqin Fu to the author list.

Yours sincerely,

Songbai Liu

Guizhou Medical University

------------------ 原始邮件 ------------------
发件人: "潘耀振" <panyaozhen@gmc.edu.cn>;
发送时间: 2022 年 4 月 26 日(星期二) 晚上 8:05
收件人: "🐬.gardenia"<1318632069@qq.com>;
主题: Change the original author list

Dear Mr Liu,

About：Manuscript Number: CDDISCOVERY-22-3681R1
Title: FAM126A Interacted with ENO1 Mediates Proliferation and Metastasis in Pancreatic Cancer via PI3K/AKT Signaling Pathway

Author Xueqin Fu will be added to the list of authors for her contributions to thesis writing and native language polishing.

Now change the original author list: Yaozhen Pan, Yongning Li, Ying Li, Jun Luo, Peng Liu, and Songbai Liu to Yaozhen Pan, Yongning Li, Ying Li, Jun Luo, Xueqin Fu, Peng Liu, and Songbai Liu. Do you agree?

Yours sincerely,

Yaozhen Pan
Guizhou Medical University
panyaozhen@gmc.edu.cn

电子邮箱 锁屏 退出 邮件全文搜索

写信

收件箱
待办邮件
草稿箱
已发送
其他文件夹
已删除
垃圾邮件
病毒文件夹

收件箱 | 回复：Cha... | 回复：Cha...

回复 | 回复全部 | 转发 | 移动到 | 标记为 | 更多 | 删除

回复：Change the original author list

.gardenia 发送给 潘耀振

2022-04-26 20:58:07

Dear Dr Pan,

About：Manuscript Number: CDDISCOVERY-22-3681R1
Title: FAM126A Interacted with ENO1 Mediates Proliferation and Metastasis in Pancreatic Cancer via PI3K/AKT Signaling Pathway

Yes, I agree to add Xueqin Fu to the author list.

Yours sincerely,

Songbai Liu

Guizhou Medical University

------------------ 原始邮件 ------------------

发件人： "潘耀振" <panyaozhen@gmc.edu.cn>;
发送时间： 2022年4月26日(星期二) 晚上8:05
收件人： " .gardenia"<1318632069@qq.com>;
主题： Change the original author list

Dear Mr Liu,

About：Manuscript Number: CDDISCOVERY-22-3681R1
Title: FAM126A Interacted with ENO1 Mediates Proliferation and Metastasis in Pancreatic Cancer via PI3K/AKT Signaling Pathway

Author Xueqin Fu will be added to the list of authors for her contributions to thesis writing and native language polishing.

Now change the original author list: Yaozhen Pan, Yongning Li, Ying Li, Jun Luo, Peng Liu, and Songbai Liu **to** Yaozhen Pan, Yongning Li, Ying Li, Jun Luo, **Xueqin Fu,** Peng Liu, and Songbai Liu. Do you agree?

Yours sincerely,

Yaozhen Pan

Guizhou Medical University

panyaozhen@gmc.edu.cn

快捷回复给所有人
